# Psycho-oncology in Korea: past, present and future

**DOI:** 10.1186/s13030-017-0097-5

**Published:** 2017-05-01

**Authors:** Hyun Jeong Lee, Kwang-Min Lee, Dooyoung Jung, Eun-Jung Shim, Bong-Jin Hahm, Jong-Heun Kim

**Affiliations:** 1 Mental Health Clinic, National Cancer Center, 323 Ilsan-ro, Ilsandong-gu, Goyang-si, Gyeonggi-do 10408 Republic of Korea; 2 Public Health Medical Service, Seoul National University Hospital, 101 Daehak-ro Jongno-gu, Seoul, 03080 Republic of Korea; 3 Department of Psychiatry, Gyeonggi Provincial Medical Center Uijeongbu Hospital, 142 Heungseon-ro, Uijeongbu-si, Gyeonggi-do 11671 Republic of Korea; 4 Department of Human Factors Engineering, Ulsan National Institute of Science and Technology, 50 UNIST-gil, Ulsan, 44919 Republic of Korea; 5 Department of Psychology, Pusan National University, 2 Busandaehak-ro 63beon-gil, Geumjeong-gu, Busan, 46241 Republic of Korea; 6 Department of Neuropsychiatry, Seoul National University Hospital, 101 Daehak-ro Jongno-gu, Seoul, 03080 Republic of Korea; 7 Department of Psychiatry and Behavioral Sciences, Seoul National University College of Medicine, 103 Daehak-ro Jongno-gu, Seoul, 03080 Republic of Korea

**Keywords:** Cancer, Psycho-oncology, Korea, History

## Abstract

**Background:**

Psycho-oncology in Korea was introduced among the circle of consultation-liaison psychiatrists, in the 1990s. For almost 25 years, the field has been developing at a steady pace as the psychosocial needs of patients with cancer continue to increase. In this study, we review the history of psycho-oncology in Korea, in a chronological order, within the domains of clinical practice, research activity, training, and public policy.

**Main body:**

Before the 1990s, patients with cancer with psychiatric comorbidities were usually taken care of by consultation-liaison psychiatrists in general hospitals. In 1993, psycho-oncology was first introduced by psychiatrists. Psychologists, nurses, and social workers have also been increasingly involved in providing psychosocial care for patients with cancer. Professionals from various disciplines began to communicate, and agreed to found the Korean Psycho-Oncology Study Group (KPOSG) in 2006, the first academic society in this field. In 2009, National Cancer Center published the “Recommendations for Distress Management in Patients with Cancer”, which are consensus-based guidelines for Korean patients. In 2014, the KPOSG was dissolved and absorbed into a new organization, the Korean Psycho-Oncology Society (KPOS). It functions as a center of development of psycho-oncology, publishing official journals, and hosting annual conferences. There are many challenges, including, low awareness of psycho-oncology, presence of undertreated psychiatric disorders in patients with cancer, shortage of well-trained psycho-oncologists, stigma, and suicide risk. It is important to improve the cancer care system to the extent that psycho-oncology is integrated with mainstream oncology. Considering the socio-cultural characteristics of Korean cancer care, a Korean model of distress management is being prepared by the KPOS.

**Conclusion:**

This article provides an overview of the development, current issues, and future challenges of psycho-oncology in Korea. Through its long journey to overcome the many barriers and stigmas of cancer and mental illnesses, psycho-oncology is now acknowledged as an essential part of integrated supportive care in cancer. Active research and international cooperation can gradually shape the Korean model of distress management.

## Background

The age-standardized rate (ASR) of cancer incidence in Korea has decreased from 303.8 per 100,000 in 2011, to 290.5 in 2013. Cancer has, however, been the most common cause of deaths in Korea since 1983; the disease accounted for 28.3% of all deaths in 2013. The age-standardized rate of cancer mortality has decreased by 2.7%, annually, from 2002 to 2013. The 5-year cancer relative survival rate has improved, from 41.2% in 1993–1995, to 69.4% in 2009–2013. The number of Korean cancer survivors has increased steadily, with 1,370,049 having been identified, as of January 1, 2014 [1].

**Table 1:** Major events of psycho-oncology in Korea

Year	Events
1993	The history, research field, and clinical application of psycho-oncology were introduced at an annual conference of the Korean Society of Psychosomatic Medicine
2000	‘National Cancer Center Act’ enacted
2003	‘Cancer Control Act’ was legislated
2006	Korean Psycho-Oncology Study Group (KPOSG) was founded
2007	An overview of psycho-oncology, research and clinical practice was presented
April, 2007	at the symposium of the Institute of Human Behavioral Medicine, Seoul National University, under the theme of ‘Psychiatric Studies: Therapy and Care’
November, 2007	The 9^th^ Cancer Control Forum sponsored with the topic of ‘Mental Suffering of Cancer Patients, How to Help?’
2009	National Cancer Center published the ‘Recommendations for Distress Management in Cancer Patients’
March, 2010	The 1^st^ KPOSG conference was held in Seoul
May, 2011	The 2^nd^ KPOSG conference was held in Seoul
May 2013	The 3^rd^ KPOSG conference was held in Sungnam
August, 2013	2013 KPOSG symposium was held in Seoul (Seoul Palace Hotel)
September, 2014	Korean Psycho-Oncology Society (KPOS) was founded The 1^st^ KPOS conference was held in Seoul
October, 2015	The 1^st^ issue of the Korean Journal of Psycho-Oncology (KJPO) was published
November, 2016	The 2^nd^ KPOS conference was held in Seoul
May, 2016	The 3^rd^ KPOS conference was held in Seoul

The diagnosis and treatment of cancer is often accompanied by psychosocial distress of patients and their families. Psychosocial distress increases the risk of psychopathology, lowers the quality of life of patients with cancer, and has a direct, or indirect negative impact on treatment and prognosis [2–4]. Because the number of patients with cancer has increased with a longer disease duration, management of their distresses has become important. Therefore, major cancer centers are focusing on distress management, from the initial treatment [5].

Psycho-Oncology, dealing with the psychological, social, and spiritual aspects of cancer, is recognized as an essential part of integrated cancer care in many developed countries. Since the World Health Organization (WHO) declared “no health without mental health” and emphasized on the importance of mental health, the need for psycho-oncological intervention has increased even further [6, 7]. Psychosocial interventions for patients with cancer are reported to improve performance indicators, including treatment outcomes and the quality of life of patients [3]. Nonetheless, owing to various reasons, the field of psycho-oncology in Korea is still perceived as a minor area of oncology.

Several studies that review the history of psycho-oncology have been published in Korea [8–10]. However, those papers were written for domestic readers, and were not focused on the history of psycho-oncology in Korea. The authors describe the history of psycho-oncology in Korea in a chronological order in the areas of clinical care, research, training, and public policy.

## Main text

### Past

The psychiatric problems of patients with cancer had been recognized in Korea long before the field of psycho-oncology was introduced, in the 1990s. Psychiatrists were interested in cancer-related psychiatric disorders, such as delirium and adjustment disorder. Consultation and liaison activities were carried out by psychiatrists mainly at general hospitals. As early as the 1970s, and through the 1980s, research papers were published in the areas of diagnosis and treatment of psychiatric disorders, truth-telling practice, hospice and end-of life care, and psychosocial interventions for patients with cancer and families. In the 1990s, hospice/palliative care, such as pain management for patients with advanced cancer, was extensively studied with the development of the hospice movement in the 1980s, and 1990s, in Korea. That was the basis of the establishment of the current hospice care system. Although studies on the quality of life, psychological symptoms of caregivers and patients with cancer, and the adaptation of the family of patients with cancer had begun to increase in the 1990s, there were limitations because of the lack of objective tools to measure such parameters.

In 1993, the concept of psycho-oncology was introduced for the first time at a symposium in the annual conference of the Korean Society of Psychosomatic Medicine [8]. The history, research field, and clinical application of the burgeoning discipline of psycho-oncology were presented by psychiatrists, and their presentations were published in the Korean Journal of Psychosomatic Medicine, in 1994.

In 1996, the Ministry of Health and Welfare of the Korean Government launched a National Cancer Control Planning Board, which embarked on the First Cancer Control 10-Year Plan (1996–2005). As the control tower of the national cancer control programs, the National Cancer Center (NCC) was established in 2000. The first decade of the 21st century witnessed a big increase of cancer centers and hospitals, in order to accommodate the increasing numbers of patients with cancer. Because issues regarding the quality of life of patients, during and after treatment, were raised, their psychosocial needs were increasingly addressed.

Although the Cancer Control Act, legislated in 2003, did not stipulate the psychosocial aspects of cancer, the importance of quality of life and palliative care were stressed in the Second 10-year Cancer Control Plan (2006–2015).

Research activities in the related fields of psycho-oncology were enriched with the establishment of the Korean Society for Hospice and Palliative Care in 1998 and the Korean Oncology Nursing Society established in 2002.

Since the 2000s, with Korean the validation of assessment tools for the symptoms and quality of life of patients with cancer and their caregivers, psycho-oncological studies using such tools have increased dramatically.

### Present

The Korean Psycho-Oncology Study Group (KPOSG) was founded in 2006, with members from multidisciplinary mental health, and related professionals. It was organized by a core group of psychiatrists, psychologists, and nurses. They shared expertise in the field of psycho-oncology through participating in monthly seminars and cooperative clinical studies. The first conference on psycho-oncology, led by members of the KPOSG, was held in April 2007. They presented topics on the overview of psycho-oncology, research, and clinical practice, at the symposium of the Institute of Human Behavioral Medicine, Seoul National University. They also organized a symposium titled, “Mental Suffering of Patients with Cancer, How to Help?” at the 9th Cancer Control Forum, sponsored by the National Cancer Control Planning Board, in November of 2007. The KPOSG has continued to hold annual academic conferences since 2010. Those conferences have attracted attention from both people in academia and the general public.

In 2008, the group conducted at reliability and validity study of the Korean version of the problem list and distress thermometer [11], one of the first distress screening tools for patients with cancer validated in the Korean language. In 2008, “Guidelines for Cancer Center Accreditation Program,” proposed by the National Cancer Center, included a section on psychosocial support for assessment (Table 1).

“The Recommendations for Distress Management in Korean Patients with Cancer” was published in 2009, by the National Cancer Center, with a grant from the National Research and Development Program for Cancer Control [11, 12]. In the recommendations, 1) the concept of distress was described, 2) distress screening based on the Modified Distress Thermometer, which combines the Korean version of the distress thermometer and the NCC Psychological Symptom Inventory (NCC-PSI), was presented [13], 3) a triage algorithm, according to the results, was presented, and 4) symptom-specific management guidelines were proposed for depression, anxiety, insomnia, and delirium, which are known disorders to have a high prevalence in patients with cancer.

With an increasing acceptance of the importance of communication in quality cancer care, NCC introduced communications skills training (CST), using the manual of the Japanese SHARE protocol translated into Korean. NCC continues to hold CST workshops for its doctors, nurses, and other employees and is trying to expand this training nationwide.

The Korean Psycho-Oncology Society (KPOS) was founded on September 26, 2014 [14]. It consists of over 80 multidisciplinary professionals, including psychiatrists, psychologists, nurses, social workers, epidemiologists, and other physicians. The KPOS hosts annual conferences, with continuing-education programs. Since 2015, the Korean Journal of Psycho-Oncology (KJPO), an official peer-reviewed journal of KPOS, is published twice a year.

### Future

The diagnosis of cancer is not considered a death sentence any more. Cancer will, however, continue to be a major health threat to people all over the world, because of the fatality and chronicity associated with the disease. Because there is an increased acceptance of the importance of quality of life in patients with cancer and families, a steady development of psycho-oncology in Korea has become possible. Awareness of psycho-oncology is still low, and the psychiatric disorders of patients with cancer tend to be under-diagnosed, and undertreated, in actual clinical practice [15, 16]. Only 10% of patients with cancer were diagnosed with comorbid psychiatric disorders, with a quarter of these patients having never had received psychiatric care [16].

According to the International Federation of Psycho-oncology Societies’ report on the disparities in psychosocial care, the development of psycho-oncology in Korea was graded as “isolated care provision’ [17], compared to the most developed level of ‘advanced integrated into mainstream service provision.”

It is important to improve the cancer care system, so that psycho-oncology is well integrated with mainstream oncology. In the Third National Cancer Control Plan (2016–2020), the scope of cancer care has been expanded, to include the quality of life of cancer survivors. The Korean government is planning to establish 13 regional integrated supportive care centers in provinces, for cancer survivors living in the community, by 2020. Those centers are going to provide comprehensive supportive care, including psychosocial service, rehabilitation, and nutritional counseling. Korean psycho-oncologists are moving toward integrated cancer care that incorporates psychosocial care as an essential component of patient care.

Although the needs for mental health professionals in the field of psycho-oncology are increasing, there is a shortage of well-trained psycho-oncologists in Korea. The Advanced Program for Psycho-Oncology (APPO), developed by NCC in 2015, is the first course to train psycho-oncologists. It is a 12-week program for psychiatrists, psychologists, social workers, and other healthcare professionals interested in psycho-oncology. Further development and expansion of training programs for psycho-oncologists would help expand the number of experts in psycho-oncology in Korea.

The effectiveness of psychosocial interventions should be proved further in order to raise awareness of psycho-oncology. Large-scale clinical studies need to be done to support the biopsychosocial model for cancer treatment.

Cancer stigma often causes psychosocial distress. According to a national survey in Korea, more than half of the public had negative attitudes, stereotypes, and discriminating attitudes toward patients with cancer in spite of clinical progress and improved survivorship [18]. A recent study in Korea reported that over 30% of Korean cancer survivors had negative attitudes toward cancer, and held stereotypical views of themselves. Furthermore, about 10% of the patients with cancer experienced social discrimination due to cancer [19]. Stigma of mental illness is a barrier for patients with cancer that needs psychiatric or psychosocial interventions. Patients with cancer with psychological problems therefore face even greater stigma. This situation should be taken into consideration in clinical practice.

Cancer is an illness that increases the risk of suicide in Asian countries [20–22]. One epidemiological study found that the suicide rate among Korean patients with cancer was approximately twice that of the general Korean population [23]. Suicide risk was higher in patients with cancers that have a poor prognosis, especially within the first year of diagnosis [23, 24]. Appropriate psycho-oncological intervention could reduce the suicide risk of patients.

With the development of information and communication technology, e-health and m-health are emerging. In psycho-oncology, there have been various studies to improve self-management and support patients with cancer, using mobile applications [25], tablet PCs [26] and touch-screen computers installed at kiosks [27]. More studies using information and communication technology will be conducted in the future.

Psycho-oncological knowledge can be universally applied, and it is beyond race, culture, and socioeconomic status; however, it is necessary to consider the socio-cultural specificity of each country. Considering the socio-cultural characteristics of Korean cancer care, a Korean model of distress management is being prepared by the KPOS, with a revision in the recommendation published in 2008.

The KPOS, as the only academic group of psycho-oncology in Korea, should tighten the cooperation with the International Psycho-Oncology Society (IPOS) and other academic communities in the world.

International cooperation with many East-Asian experts who share similar cultural and healthcare system backgrounds would help with the promotion of psycho-oncology in Korea.

## Conclusions

The introduction of psycho-oncology to Korea was relatively late, and its growth was not fast enough, compared to Western countries and Japan. There has, however, been a steady development of psycho-oncology, with a successful launching of a national academic society and growing numbers of enthusiastic professionals in Korea. This article provided a brief overview of the development, current issues, and future challenges of psycho-oncology in Korea. Psycho-oncology should be an integral part of integrated supportive care for cancer in Korea.

## Abbreviation

APPO: Advanced Program for Psycho-Oncology
ASR: Age-standardized rate
CST: Communications skills training
IPOS: International Psycho-Oncology Society
KJPO: Korean Journal of Psycho-Oncology
KPOS: Korean Psycho-Oncology Society
KPOSG: Korean Psycho-Oncology Study Group
NCC: National Cancer Center
NCC-PSI: National Cancer Center - Psychological Symptom Inventory
WHO: World Health Organization

## References

[1] Oh CM, Won YJ, Jung KW, Kong HJ, Cho H, Lee JK (2016). Cancer statistics in Korea: incidence, mortality, survival, and prevalence in 2013. Cancer Res Treat.

[2] Zabora J, BrintzenhofeSzoc K, Curbow B, Hooker C, Piantadosi S (2001). The prevalence of psychological distress by cancer site. Psychooncology.

[3] Antoni MH (2013). Psychosocial intervention effects on adaptation, disease course and biobehavioral processes in cancer. Brain Behav Immun.

[4] Dolbeault S, Szporn A, Holland JC (1999). Psycho-oncology: where have we been? Where are we going?. Eur J Cancer.

[5] Bruera E, Hui D (2010). Integrating supportive and palliative care in the trajectory of cancer: establishing goals and models of care. J Clin Oncol.

[6] Prince M, Patel V, Saxena S, Maj M, Maselko J, Phillips MR (2007). No health without mental health. Lancet.

[7] Clarke DM (2010). No cancer health without mental health. Med J Aust.

[8] Lee C (1994). Psycho-oncology : A Historical Review. Korean J Psychosomatic Med.

[9] Hahm BJ, Shim EJ, Kim HK, Kim JH (2007). History and Current Status of Psycho-Oncology. J Korean Neuropsychiatr Assoc.

[10] KM Lee, DJ, EJ Shim, BJ Hahm. Understanding of Psycho-Oncology: Historical Background, Current Status and Future Perspectives. Korean J Psycho-Oncol. 2015;1(1):1–10

[11] Shim EJ, Shin YW, Jeon HJ, Hahm BJ (2008). Distress and its correlates in Korean cancer patients: pilot use of the distress thermometer and the problem list. Psychooncology.

[12] Yu ES, Shim EJ, Kim HK, Hahm BJ, Park JH, Kim JH (2012). Development of guidelines for distress management in Korean cancer patients. Psychooncology.

[13] Shim EJ, Hahm BJ, Yu ES, Kim HK, Cho SJ, Chang SM, et al. Development and validation of the National Cancer Center Psychological Symptom Inventory. Psychooncology. 2016; doi:10.1002/pon.4277. 10.1002/pon.4277 27605487

[14] Korean Psycho-Oncology Society. http://www.kpos-society.org. Accessed 7 Jan 2017.

[15] Kang JI, Sung NY, Park SJ, Lee CG, Lee BO (2014). The epidemiology of psychiatric disorders among women with breast cancer in South Korea: analysis of national registry data. Psychooncology.

[16] Lee BO, Choi WJ, Sung NY, Lee SK, Lee CG, Kang JI (2015). Incidence and risk factors for psychiatric comorbidity among people newly diagnosed with cancer based on Korean national registry data. Psychooncology.

[17] Grassi L, Fujisawa D, Odyio P, Asuzu C, Ashley L, Bultz B (2016). Disparities in psychosocial cancer care: a report from the International Federation of Psycho-oncology Societies. Psychooncology.

[18] Cho J, Smith K, Choi EK, Kim IR, Chang YJ, Park HY (2013). Public attitudes toward cancer and cancer patients: a national survey in Korea. Psychooncology.

[19] Cho J, Choi EK, Kim SY, Shin DW, Cho BL, Kim CH (2013). Association between cancer stigma and depression among cancer survivors: a nationwide survey in Korea. Psychooncology.

[20] Akechi T, Kugaya A, Okamura H, Nakano T, Okuyama T, Mikami I (1999). Suicidal thoughts in cancer patients: clinical experience in psycho-oncology. Psychiatry Clin Neurosci.

[21] Akechi T, Okamura H, Yamawaki S, Uchitomi Y (2001). Why do some cancer patients with depression desire an early death and others do not?. Psychosomatics.

[22] Park JY, Moon KT, Chae YM, Jung SH (2008). Effect of sociodemographic factors, cancer, psychiatric disorder on suicide: gender and age-specific patterns. Journal of preventive medicine and public health =. Yebang Uihakhoe chi.

[23] Ahn E, Shin DW, Cho SI, Park S, Won YJ, Yun YH (2010). Suicide rates and risk factors among Korean cancer patients, 1993–2005. Cancer Epidemiol Biomark Prev.

[24] Ahn MH, Park S, Lee HB, Ramsey CM, Na R, Kim SO (2015). Suicide in cancer patients within the first year of diagnosis. Psychooncology.

[25] Hee Jun Kim M-sK, Jae-hyun Tae, Song-Ee Park, Sun Mi Kim, Hee-Chul Shin, Dong Hyun Han: Mobile game for management in metastatic breast cancer patients receiving cytotoxic chemotherapy. In: 2016 ASCO Annual Meeting. Chicago: 2016 ASCO Annual Meeting Proceedings; 2016.

[26] Lee JY, Park HY, Jung D, Moon M, Keam B, Hahm BJ (2014). Effect of brief psychoeducation using a tablet PC on distress and quality of life in cancer patients undergoing chemotherapy: a pilot study. Psychooncology.

[27] Lee JY, Jung D, Kim WH, Lee HJ, Noh DY, Hahm BJ (2016). Correlates of oncologist-issued referrals for psycho-oncology services: what we learned from the electronic voluntary screening and referral system for depression (eVSRS-D). Psychooncology.

